# Fixing a Hole: Preventing Pneumococcal Pneumonia by Vaccination

**DOI:** 10.3389/fimmu.2016.00349

**Published:** 2016-09-13

**Authors:** Ger T. Rijkers

**Affiliations:** ^1^Science Department, University College Roosevelt, Middelburg, Netherlands; ^2^Laboratory for Medical Microbiology and Immunology, St. Antonius Hospital, Nieuwegein, Netherlands

**Keywords:** *Streptococcus pneumoniae*, polysaccharide, vaccination, infants, elderly, inflammation

The immune system of the human newborn is capable of responding to every microorganism or one of its components it encounters. In fact that is not true, there is one exception. The immune system of newborns is unable to respond to the capsular polysaccharides of bacteria such as *Streptococcus pneumoniae*. Because the immune system does not respond, *S. pneumoniae* can cause diseases such as pneumonia, meningitis, bacteremia, and otitis media, and children up to the age of 2, maybe even 5 years (1, 2). From personal experience, I recall an episode of otitis media during the summer of 1955 as being extremely painful. I cried as loud and long as I could, but the substitute for our own family doctor, who was on vacation, was not impressed by the expression of my suffering. Fortunately, our own doctor returned just in time so that penicillin saved me and I survived. Otitis media is the most common disease caused by *S. pneumoniae*, but pneumonia is the deadliest. Every year, on a worldwide scale, an estimated one and a half million children below the age of 5 years die from the consequences of pneumonia (2). Why does the immune system not respond to these bacteria, apparently not even try to? Is there a hole in the immune system that lets these bacteria in, and although the holes could be rather small, the consequences could be severe. The cell-biological answer to this why question could be rather simple: the B lymphocytes of the newborn do not express the type 2 complement receptor on their cell surface (3, 4). This receptor is crucial for providing a costimulatory signal to the B lymphocyte, which otherwise cannot respond to polysaccharides (5). At the age of approximately 2 years, the B lymphocytes start to express this receptor, acquire the capacity to respond, and produce antibodies to the polysaccharide, and from that point on the child is protected (6, 7). But keep in mind that nobody is really sure whether all pneumococcal serotypes would activate B lymphocytes in a similar way. This, however, still does not quite answer the why question. Why does it take so long to develop responsiveness, and, what is the evolutionary benefit? It could be hypothesized that *S. pneumoniae* can colonize the mucosal surfaces of the upper respiratory tract of young children because the immune system does not react (8). By doing so, it occupies all available niches, leaving no place for other bacterial pathogens. This will protect young children against many other infectious diseases, but the pay-off for this protection is the burden of pneumonia.

When an immune system is not strong enough, it can be enhanced by vaccination. For *S. pneumoniae* that does not work for young children, because a polysaccharide vaccine would not induce an immune response. If you cannot change the human host, and if you cannot change the bacterium, then the only solution left is to change the vaccine. That is exactly what has been done by Oswald Avery, back in 1929 (9). His breakthrough discovery, believed by many to be of Nobel prize-winning magnitude, was that by covalent coupling of a protein to the bacterial polysaccharide you can change the nature of the immune response to the polysaccharide. Conjugation of protein to polysaccharide is the reason, the magic trick if you want, which makes that infants can respond to the vaccine, and be protected against pneumococcal infections. This discovery by Avery was made at the right place (the Rockefeller Institute in New York) but certainly at the wrong time (namely, in 1929). During that period, it was not fashionable to study prevention of infectious diseases because it was believed that antibiotics would make such studies obsolete. The enormous success of antibiotics prompted the US Surgeon General, William Stewart, in 1969 to address the US Congress. He said it was time to “close the books on infectious diseases.” This famous quote has been repeated innumerable times to underscore how wrong he was. The original source of this quote however is difficult, if not impossible to trace, and therefore, it may very well be an urban legend (10). Closer to immunology, Sir McFarlane Burnet wrote in 1962: “One can think of the middle of the twentieth century as the end of one of the most important social revolutions in history, the virtual elimination of the infectious diseases as a significant factor in social life” (11). If being wrong could be graded, Burnet would deserve an A^+^.

Because of the above illustrations of the widespread belief of the supremacy of man over microbe, it took almost 50 years before the principles of Oswald Avery were rediscovered and polysaccharide conjugate vaccines were developed and became available (12). The story of pneumococcal vaccines is complicated because for the immune system, *S. pneumoniae* is not one single bacterium but at least 93 recognized different ones (13), such as cellophane flowers of yellow and green. That is because the capsular polysaccharide comes in different compositions, and an ideal vaccine thus should be composed of all these 93 polysaccharides. Fortunately, the top 13 polysaccharides cover most (close to 80%) of all pneumococcal infections. This top 13 is the top 13 of pneumococcal infections in White, American children (10). Approximately 20 years ago today, the pneumococcal conjugate vaccine also was introduced in Europe and the Netherlands. The coverage of the 13-valent vaccine in other parts of the world, such as South-East Asia and Africa, unfortunately is substantially lower (14). We are studying which pneumococcal serotypes cause disease in children from Bangladesh, a sub-study of a large global initiative (15). The ultimate goal is to identify the pneumococci and other microorganisms that cause childhood pneumonia and subsequently design and implement effective vaccines that can improve survival rates of children across the globe.

Not only during childhood but also later in life, usually much later in life, you will meet *S. pneumoniae* again. “You’ll be older too, and if you say the word, I could stay with you” could be the motto of the pneumococci. William Osler touched upon this subject in The Principles and Practice of Medicine, a textbook which was first published in 1892 and remained in print for 55 years and 16 editions.^1^ In the first edition, in the chapter on Diseases of the Respiratory System, Osler describes on page 526 the prognosis of pneumonia: “In children and in healthy adults the outlook is good. In the debilitated, in drunkards, and in the aged the chances are against recovery. So fatal is it in the latter class that it has been termed the natural end of the old man.” In the third edition of his book, this last sentence has been rephrased. It now reads as “Pneumonia may well be called the friend of the aged. Taken off by it in an acute, short, not often painful illness, the old man escapes those ‘cold gradations of decay’ so distressing to himself and to his friends.” Since that time, pneumonia has been termed the old man’s friend, although it sacrificed most of our lives. It is ironical that in 1919, Osler himself died after a protracted pneumonia. But that was more than 20 years ago, in fact almost 100 years ago, 10 years before Fleming discovered penicillin. The 13-valent pneumococcal conjugate vaccine has shown to be effective in elderly (16, 17) and hopefully will be implemented in the near future.

Clearly, because of the fact that 93 serotypes of *S. pneumoniae* exist, a vaccine with 100% coverage will be virtually impossible to achieve. Thus, although prevention would be preferred, there will always be the need for treatment of pneumococcal infections. Patients with pneumococcal pneumonia now can be treated with antibiotics, penicillin, or other antibiotics, and most of them survive. The mortality rate of pneumococcal pneumonia has dropped from 50% to below 5% (18). After all, there may have been some truth in the statement of Surgeon General William Stewart. Most of the clinical symptoms of the pneumonia now in fact are caused by an overactive immune system. That is because the immune system has no way of knowing that antibiotics have already killed the bacteria, shortly after the start of the infection. The fragments of killed bacteria are an even stronger stimulus for the immune system (19). Is this really to the benefit of Mr. Kite, to the Hendersons, or any other patient with pneumococcal pneumonia? Can one desire too much of a good thing, is what Rosalind asks Orlando in Shakespeare’s As You Like It (1600).^2^ Apparently yes, you can have too much of a good immune response. Therefore, it is attractive to postulate that dampening the immune response, thereby reducing inflammation, during pneumococcal pneumonia could have a clinical beneficial effect. Treatment with anti-inflammatory drug, such as dexamethasone indeed, reduces the length of hospital stay and improves quality of life afterward (20, 21). The combined effect of optimal vaccination strategies and balanced antibiotic and anti-inflammatory treatment thus has dramatically improved the outlook for prevention and recovery of pneumococcal infection, both for the very young and the very old. It is getting better all the time.

## Author Notes

This opinion paper is based on the inaugural lecture of GT Rijkers, the full text can be found at http://www.gtrijkers.nl/SurvivingLife_Web.pdf. This opinion paper includes various quotes from the biomedical literature, which are specified in the list of references. Apart from the title, it also contains additional quotes from the Sergeant Peppers album of the Beatles, of which the full lyrics can be found at http://www.thebeatles.com/album/sgt-peppers-lonely-hearts-club-band.

## Author Contributions

The author confirms being the sole contributor of this work and approved it for publication.

## Conflict of Interest Statement

The author declares that the research was conducted in the absence of any commercial or financial relationships that could be construed as a potential conflict of interest.

## Footnotes

^1^http://archive.org/details/principlesandpr00mccrgoog

^2^http://shakespeare.mit.edu/asyoulikeit/full.html

## References

[1] Paton JC Toogood IR Cockington RA Hansman D Antibody response to pneumococcal vaccine in children aged 5 to 15 years. Am J Dis Child (1993) 140(2):135–8. 3946323 10.1001/archpedi.1986.02140160053031

[2] Walker CL Rudan I Liu L Nair H Theodoratou E Bhutta ZA Global burden of childhood pneumonia and diarrhoea. Lancet (2013) 381:1405–16. 10.1016/S0140-6736(13)60222-6 23582727 PMC7159282

[3] Griffioen AW Franklin SW Zegers BJ Rijkers GT. Expression and functional characteristics of the complement receptor type 2 on adult and neonatal B lymphocytes. Clin Immunol Immunopathol (1993) 69(1):1–8. 8403536 10.1006/clin.1993.1142

[4] Mitsuyoshi JK Hu Y Test ST. Role of complement receptor type 2 and endogenous complement in the humoral immune response to conjugates of complement C3d and pneumococcal serotype 14 capsular polysaccharide. Infect Immun (2005) 73(11):7311–6. 10.1128/IAI.73.11.7311-7316.2005 16239528 PMC1273882

[5] Pozdnyakova O Guttormsen HK Lalani FN Carroll MC Kasper DL. Impaired antibody response to group B streptococcal type III capsular polysaccharide in C3- and complement receptor 2-deficient mice. J Immunol (2003) 170(1):84–90. 10.4049/jimmunol.170.1.84 12496386

[6] Timens W CD21. J Biol Regul Homeost Agents (2000) 4(4):292–4. 11215823

[7] Lee CJ Banks SD Li JP. Virulence, immunity, and vaccine related to *Streptococcus pneumoniae*. Crit Rev Microbiol (1991) 18:89–114. 10.3109/10408419109113510 1930677

[8] Rijkers GT van Mens SP van Velzen-Blad H What do the next 100 years hold for pneumococcal vaccination? Expert Rev Vaccines (2010) 9(11):1241–4. 10.1586/erv.10.127 21087102

[9] Avery O Goebel WF Chemo-immunological studies on conjugated carbohydrate-proteins. II. Immunological specificity of synthetic sugar protein antigens. J Exp Med (1929) 50:533. 19869645 10.1084/jem.50.4.533 PMC2131643

[10] Spellberg B Taylor-Blake B. On the exoneration of Dr. William H. Stewart: debunking an urban legend. Infect Dis Poverty (2013) 2:3. 10.1186/2049-9957-2-3 23849720 PMC3707092

[11] Burnet M Natural History of Infectious Disease. Cambridge, UK: Cambridge University Press (1962).

[12] Whitney CG Klugman KP. Vaccines as tools against resistance: the example of pneumococcal conjugate vaccine. Semin Pediatr Infect Dis (2004) 15(2):86–93. 10.1053/j.spid.2004.01.011 15185191

[13] Geno KA Gilbert GL Song JY Skovsted IC Klugman KP Jones C Pneumococcal capsules and their types: past, present, and future. Clin Microbiol Rev (2015) 28(3):871–99. 10.1128/CMR.00024-15 26085553 PMC4475641

[14] Rodgers GL Arguedas A Cohen R Dagan R. Global serotype distribution among *Streptococcus pneumoniae* isolates causing otitis media in children: potential implications for pneumococcal conjugate vaccines. Vaccine (2009) 27(29):3802–10. 10.1016/j.vaccine.2009.04.021 19446378

[15] Levine OS O’Brien KL Deloria-Knoll M Murdoch DR Feikin DR DeLuca AN The pneumonia etiology research for child health project: a 21st century childhood pneumonia etiology study. Clin Infect Dis (2012) 54(Suppl 2):S93–101. 10.1093/cid/cir1052 22403238 PMC3297546

[16] Bonten MJ Huijts SM Bolkenbaas M Webber C Patterson S Gault S Polysaccharide conjugate vaccine against pneumococcal pneumonia in adults. N Engl J Med (2015) 372(12):1114–25. 10.1056/NEJMoa1408544 25785969

[17] van Werkhoven CH Bonten MJ. The community-acquired pneumonia immunization trial in adults (CAPiTA): what is the future of pneumococcal conjugate vaccination in elderly? Future Microbiol (2015) 10(9):1405–13. 10.2217/fmb.15.80 26347153

[18] Watson DA Musher DM Jacobson JW Verhoef J A brief history of the pneumococcus in biomedical research: a panoply of scientific discovery. Clin Infect Dis (1993) 17(5):913–24. 10.1093/clinids/17.5.913 8286641

[19] Steel HC Cockeran R Anderson R Feldman C. Overview of community-acquired 407 pneumonia and the role of inflammatory mechanisms in the immunopathogenesis of severe 408 pneumococcal disease. Mediators Inflamm (2013) 2013:490346. 10.1155/2013/490346 24453422 PMC3886318

[20] Meijvis SC Hardeman H Remmelts HH Heijligenberg R Rijkers GT van Velzen-Blad H Dexamethasone and length of hospital stay in patients with community-acquired pneumonia: a randomised, double-blind, placebo-controlled trial. Lancet (2011) 377(9782):2023–30. 10.1016/S0140-6736(11)60607-7 21636122

[21] Revest M Egmann G Chapron A Jouneau S Tattevin P Adjuvant corticosteroids for patients hospitalized with community-acquired pneumonia: is it time? J Thorac Dis (2016) 8(5):E288–91. 10.21037/jtd.2016.03.34 27162684 PMC4842784

